# 9β Polymorphism of the Glucocorticoid Receptor Gene Appears to Have Limited Impact in Patients with Addison’s Disease

**DOI:** 10.1371/journal.pone.0086350

**Published:** 2014-01-23

**Authors:** Ian Louis Ross, Collet Dandara, Marelize Swart, Miguel Lacerda, Desmond Schatz, Dirk Jacobus Blom

**Affiliations:** 1 Division of Endocrinology, Department of Medicine, University of Cape Town, Cape Town, South Africa; 2 Department of Human Genetics, University of Cape Town, Cape Town, South Africa; 3 Department of Statistical Sciences, University of Cape Town, Cape Town, South Africa; 4 Department of Paediatrics, College of Medicine, University of Florida, Gainesville, Florida, United States of America; 5 Division of Lipidology, Department of Medicine, University of Cape Town, Cape Town, South Africa; IPO, Inst Port Oncology, Portugal

## Abstract

**Background:**

Addison’s disease (AD) has been associated with an increased risk of cardiovascular disease. Glucocorticoid receptor polymorphisms that alter glucocorticoid sensitivity may influence metabolic and cardiovascular risk factors in patients with AD. The 9β polymorphism of the glucocorticoid receptor gene is associated with relative glucocorticoid resistance and has been reported to increase the risk of myocardial infarction in the elderly. We explored the impact of this polymorphism in patients with AD.

**Materials and Methods:**

147 patients with AD and 147 age, gender and ethnicity matched healthy controls were recruited. Blood was taken in a non-fasted state for plasma lipid determination, measurement of cardiovascular risk factors and DNA extraction.

**Results:**

Genotype data for the 9β polymorphism was available for 139 patients and 146 controls. AD patients had a more atherogenic lipid profile characterized by an increase in the prevalence of small dense LDL (p = 0.003), increased triglycerides (p = 0.002), reduced HDLC (p<0.001) an elevated highly sensitive C-reactive protein (p = 0.01), compared with controls. The 9β polymorphism (at least one G allele) was found in 28% of patients and controls respectively. After adjusting for age, gender, ethnicity, BMI and hydrocortisone dose per metre square of body surface area in patients, there were no significant metabolic associations with this polymorphism and hydrocortisone doses were not higher in patients with the polymorphism.

**Conclusions:**

This study did not identify any associations between the 9β polymorphism and cardiovascular risk factors or hydrocortisone dose and determination of this polymorphism is therefore unlikely to be of clinical benefit in the management of patients with AD.

## Introduction

Addison’s disease (AD) has been associated with an up to two-fold increased mortality rate from cardiovascular disease [1], [2]. Supra-physiological hydrocortisone replacement may cause multiple side-effects including multiple metabolic disturbances [3]. Ross et al, previously reported that patients with AD had higher triglycerides (TG), lower high density lipoprotein cholesterol (HDLC), a preponderance of small dense low density lipoprotein (LDL) particles and raised high sensitivity C-reactive protein (hs-CRP) compared with healthy controls. These findings may in part account for the increased rates of cardiovascular disease seen in patients with AD [4].

The human glucocorticoid receptor (GCR) gene is located on chromosome 5 (5q31) and has nine exons [5]. There are polymorphisms of varying functional significance throughout the GCR gene. The BclI and N363S polymorphisms have been reported to increase cortisol sensitivity [6]–[8], whereas the ER22/23EK polymorphism is associated with glucocorticoid resistance [9]. Ross et al previously studied the effects of the above polymorphisms in patients with AD and found that the ER22/23EK polymorphism was counter-intuitively associated with an increased BMI, but lower LDL-cholesterol. The mean hydrocortisone dose was higher in patients with either the ER22/23EK or N363S polymorphism [4]. We now report the results of our analysis of the 9β polymorphism in the same cohort.

The 9β polymorphism, located in the 3′untranslated region of the GCR, is postulated to increase the stability of the GCRβ splice variant, which acts as a dominant negative on the functional wild-type GCRα [10]. The 9β polymorphism has been reported to cause relative glucocorticoid resistance [11].

The 9β polymorphism has also been associated with an increased risk of myocardial infarction, especially in an elderly sub-group. It was speculated that the higher risk of ischaemic heart disease may be due to reduced cortisol suppression of the pro-inflammatory system. Patients that were homozygous for this polymorphism had increased intima media thickness, increased hs-CRP, increased interleukin 6 and an almost threefold increased risk of cardiovascular disease [11]. We therefore explored the effect of the 9β polymorphism on AD hypothesizing that it may be associated with a need for higher replacement doses of hydrocortisone and increased inflammatory markers as previously reported.

## Materials and Methods

### Subjects and Methods

This study was approved by the University of Cape Town Human Research and Ethics Committee, which subscribes to the latest version of the Declaration of Helsinki and all participants gave written informed consent. A total of 147 patients with AD and 147 age, gender and ethnicity matched healthy controls were recruited at a volunteer blood donor clinic. Patients and control subjects reported their own ethnicity. The patients were recruited from the South African national database of AD, in which all medical practitioners were asked to register patients with AD in their care. Clinical data were extracted from patients’ notes and patient interviews. Blood was taken in a non-fasted state for plasma lipid determination, measurement of cardiovascular disease risk markers and DNA extraction.

### Hydrocortisone Dose

Hydrocortisone doses were modified by the patients’ treating physicians based on symptoms that were compatible with either cortisol deficiency or excess. There were no standard protocols to modify replacement doses. The majority of patients received a dose of approximately 20 mg of hydrocortisone per day, irrespective of body weight. The authors had no influence on hydrocortisone dosing, since this was an observational study. Only well patients, on stable doses of hydrocortisone replacement for a minimum of three months were enrolled in this study. Concomitant medication was carefully reviewed in order to ensure that the hydrocortisone dose was not influenced by drugs that alter hydrocortisone metabolism.

### Assays for Lipids, Lipoproteins and Markers of Cardiovascular Risk

Assays for TG, TC, non-esterified fatty acids (NEFA) and random blood glucose (RBG) were performed using commercially available enzymatic kits. We used kits from Kat Medical (Roodepoort, Gauteng, South Africa) to measure triglycerides, cholesterol and glucose and a kit from Roche (Roche diagnostics Corporation, Indianapolis, Indiana, USA) for NEFA. HDLC was measured following the first step in the Gidez assay, which yields HDLC in the supernatant of a heparin-Mn precipitation of apoB-containing lipoproteins [12]. LDL particle size was measured by non-denaturing gradient gel electrophoresis [13]. LDLC was calculated by the Friedewald equation [14].

Highly sensitive-C reactive protein (hs-CRP) was measured using an immuno-turbidometric assay (Roche Diagnostics, GmbH, Mannheim, Germany) demonstrating a coefficient of variation of 4% and 3% at serum concentrations of 1 mg/L and 15 mg/L, respectively. We calculated 10 year coronary heart disease risk using an older version of the Framingham risk algorithm that still includes diabetes as a risk factor so that comparisons could be made across the entire cohort [15]. Diabetes is currently regarded as a secondary prevention equivalent for cardiovascular disease and is no longer included as a risk factor in the Framingham algorithm [16].

### DNA Extraction and Genotyping for the GCR 9β Polymorphism

Genomic DNA was extracted using a Promega wizard DNA extraction kit (Wisconsin, USA), according to the manufacturer’s protocol [17] and stored at −20°C until needed. The genomic DNA was used to amplify a section of the GCR gene which enabled the detection of the GCR 9β polymorphism (i GCR c.*3833A>G) in both patients and healthy controls, using PCR, followed by restriction fragment length polymorphism analysis (PCR-RFLP) according to the method by Donn et al. [18] Forward and reverse primers used in the PCR amplification were 5′-AGTGTCTTTTTACCTACGC-3′ and 5′-ATGTTTCTCCATATTTGGC-3′, respectively. Each PCR amplification reaction contained 100–200 ng genomic DNA, 1 X PCR buffer, 1.5 mM MgCl_2_, 200 µM deoxyribonucleotide triphosphates (dNTPs), 0.4 µM of each primer and 1 U *Taq* DNA polymerase (Fermentas, Thermo Scientific, Waltham, MA, USA) in a final volume of 25 µl. PCR amplification conditions included an initial denaturation step at 94°C for 3 min, followed by 35 cycles of denaturation at 94°C for 30 sec, annealing at 52°C for 30 seconds and elongation at 72°C for 30 seconds, followed by a final extension step at 72°C for 10 min. PCR products were digested using *SwaI* (Fermentas Thermo Scientific, Waltham, MA, USA) restriction enzyme. Restriction of the 172 bp PCR product in the presence of the GCR c.*3833A allele produced 97 bp and 75 bp fragments, while the presence of c.*3833G allele resulted in a 172 bp fragment. PCR digestion fragments were separated on an 2% agarose gel, visualised on UV in the presence of ethidium bromide staining and compared to a standard marker (100 bp plus marker (Fermentas)). Genotyping results using PCR-RFLP were further confirmed by sequencing two DNA samples for each of the genotypes, c.3833G/G, G/A and A/A, respectively. Genotyping quality was verified using the Hardy-Weinberg Equilibrium (HWE) calculations on the controls and patients.

### Statistical Methods

The clinical characteristics of patients and controls were compared using the Wilcoxon rank sum test for continuous traits (such as age and BMI) and Fisher’s exact test for discrete traits (such as gender and ethnicity). Associations with the 9β polymorphism were assessed by regressing each clinical characteristic against genotype and testing the null hypothesis of equal regression coefficients after adjusting for age, gender, ethnicity, BMI, and hydrocortisone dose per square meter of body surface area in patients. The results without adjusting for these potential confounders did not differ substantively. Standard linear regression models were used for continuous characteristics (using a logarithmic transformation to remove skewness where necessary) and logistic regression models were employed for dichotomous characteristics. Differences in the nature of the association between each clinical characteristic and the 9β genotype between patients and controls were assessed by testing for a significant interaction term between genotype and treatment group in the regression models. An extreme measurement of 31.2 mlU/L for the thyroid stimulating hormone (TSH) was removed from the analyses. All analyses were performed in the R Language and Environment for Statistical Computing [19]. P-values below 0.05 were regarded as statistically significant.

## Results

We recruited 147 patients with AD from the South African national AD database and 147 healthy controls from a volunteer blood donor clinic. We matched controls for gender, ethnicity and age whenever possible. Genotype data for the 9β polymorphism was available for 139 patients and 146 controls and these subjects were included in our further analyses. The underlying aetiology of AD was autoimmune in 90 (65%), tuberculosis in 10 (7%), X-linked adrenal hypoplasia in 8 (6%), adrenoleukodystrophy in 5 (4%), ACTH resistance in 2 (1%) and 24 (17%) were considered idiopathic.

The patients and controls were well matched for gender and ethnicity, but the patients as a group were older and exhibited a lower BMI. A more atherogenic lipid profile was seen in the patients, identified by a greater proportion with small dense LDL, increased TG, reduced HDLC and elevated hs-CRP levels. Nineteen (13%) and 0 (0%) patients and controls received lipid-lowering therapy respectively (Table 1). In addition NEFA were lower in patients compared to controls.

**Table 1:** Clinical characteristics of Addison’s disease patients and controls.

Clinical characteristics	Patients *N* = 139	Controls *N* = 146	*p*-value Patients versus controls*
Age (IQR) years	46.0 (33.25–60.75)	41.0 (33–53)	0.04373
Gender *N* (%)			1.00
female	86 (0.61)	90 (0.68)	
male	53 (0.39)	56 (0.32)	
Ethnicity			0.995
White	89 (0.64)	96 (0.66)	
Mixed ancestry	34 (0.24)	34 (0.23)	
Asian	5 (0.04)	5 (0.03)	
Black	11 (0.08)	11 (0.08)	
BMI (IQR) kg/m^2^	24.8 (22.18–30.25)	26.4 (24.1–31.2)	0.01
TG (IQR) mmol/L	1.705 (1.108–2.61)	1.37 (0.96–2.11)	0.002
TC (IQR) mmol/L	5.69 (4.66–6.695)	5.69 (4.97–6.42)	0.907
HDLC (IQR) mmol/L	0.78 (0.5325–1.09)	1.08 (0.935–1.27)	<0.001
LDLC (IQR) mmol/L	4.17 (3.008–4.93)	3.7 (3.2–4.4)	0.236
Small dense LDL n/N (%)	17/118 (0.144)	5/141 (0.035)	0.003
NEFA µmol/L	329 (137–654)	467 (324.5–646)	0.001
Lipid lowering n/N (%)	19/139 (0.137)	0/0 (NA)	NA
hs–CRP mg/L	2.2 (1–6.2)	1.5 (0.635–3.3)	0.0107

*N*: number, NEFA: non-esterified fatty acids, IQR: interquartile range, SD: standard deviation.

* Significant at the 5% level, NA: not applicable, n/N: total number of patients found with small dense LDL or using lipid-lowering therapy/total number of patients or controls.

The genotyping quality was verified using the Hardy Weinberg equilibrium (HWE) calculations on the controls. The genotype did not deviate from HWE in either the controls (p = 0.441) or the patients (p = 0.482). There was significant linkage disequilibrium between the 9β (G allele) and both BclI (G allele); [p<0.001] and ER22/23EK (GAA AAG allele); [p = 0.001] polymorphisms, respectively.

### Dosage of Hydrocortisone Replacement

The median (interquartile range, IQR) dose of hydrocortisone adjusted for body weight was 0.33 (0.25–0.45) mg/kg. Most patients were maintained on doses between 0.2 to 0.4 mg/kg, but there were occasional patients taking very high doses.

We examined the relationships between the metabolic parameters and the 9β polymorphism. Control subjects carrying the 9β polymorphism were older than wild type subjects. Among the controls, the median LDLC was lowest for those with the 9β polymorphism wild type and lower for the heterozygous relative to those who were homozygotes for the 9β allele (table 2). Interestingly the opposite trend was observed for patients. Although this interaction is statistically significant at per-comparison level (p = 0.020), it does not withstand adjustment for multiple testing. The remaining results are shown in Table 2. We reanalyzed the data after merging homozygous and heterozygous subjects into a single group. The results of this analysis were not substantively different from our original analysis.

**Table 2:** Clinical characteristics associated with the 9β polymorphism in healthy controls and AD patients.

Clinical characteristics	Homozygous G/G genotype	Heterozygous G/A genotype	Wild Type A/A genotype	Controls	Patients	Interaction^1^
Group *N* (%)						0.581
Controls	5 (0.03)	36 (0.25)	105 (0.72)			
Patients	2 (0.01)	37 (0.27)	100 (0.72)			
BMI (IQR) kg/m^2^				0.617	0.674	0.805
Controls	30.7 (27.4–31.5)	27.4 (23.7–32.0)	26.3 (24.1–31.1)			
Patients	28.2 (27.1–29.3)	24.1 (22.7–29.0)	24.8 (22.0–30.2)			
Hypertension Patients	0 (0.00)	8 (0.38)	13 (0.62)		0.165	
Diabetes Patients	(0.00)	7 (0.41)	10 (0.59)		0.259	
Triglycerides (IQR) mmol/L				0.323	0.499	0.412
Controls	1.12 (1.12–1.37)	1.67 (0.93–2.22)	1.32 (0.98–1.89)			
Patients	2.77 (1.89–3.64))	1.82 (1.29–2.71)	1.65 (1.10–2.56)			
Total cholesterol (SD) mmol/L				0.369	0.754	0.290
Controls	6.80 (1.84)	5.89 (1.22)	5.67 (1.24)			
Patients	5.43 (3.40)	5.83 (1.28)	5.74 (1.59)			
HDLC (IQR) mmol/L				0.978	0.716	0.193
Controls	1.16 (1.10–1.25)	1.05 (0.91–1.22)	1.09 (0.93–1.27)			
Patients	0.77 (0.77–0.77)	0.88 (0.70–1.17)	0.77 (0.44–0.99)			
LDLC (IQR) mmol/L				0.035	0.078	0.020
Controls	4.75 (3.68–6.23)	3.70 (3.30–4.18)	3.60 (3.15–4.35)			
Patients	1.79 (1.79–1.79)	3.73 (2.95–4.68)	4.28 (3.02–5.04)			
Small dense LDL				0.336	0.701	0.510
Controls	0 (0.00)	0 (0.00)	5 (1.00)			
Patients	1 (0.06)	3 (0.18)	13 (0.76)			
NEFA (IQR) µmol/L				0.740	0.704	0.689
Controls	447.5 (437.0–609.2)	486.0 (298.5–674.5)	463.0 (328.0–639.8)			
Patients	633.0 (582.5–683.5)	417.0 (192.0–635.0)	230.0 (126.2–627.2)			
Lipid-lowering therapy					0.290	
Patients	0 (0.0)	7 (0.37)	12 (0.63)			
hs-CRP (IQR) mg/L				0.743	0.485	0.769
Controls	0.89 (0.42–6.3)	1.55 (0.61–3.85)	1.50 (0.71–2.68)			
Patients	7.50 (6.90–8.10)	2.25 (0.87–8.70)	2.10 (1.05–5.00)			
Hydrocortisone daily dose (IQR) mg					0.296	
Patients	13.75 (13.12–14.38)	30.00 (20.00–30.00)	20.00 (20.00–30.00)			
Framingham risk (IQR)					0.280	
Patients	29.00 (29.00–29.00)	13.50 (5.195–21.64)	13.30 (6.50–25.70)			
TSH (IQR) mIU/L					0.543	
Patients	2.76 (2.30–3.22)	1.60 (0.53–1.87)	1.26 (0.83–2.16)			

*N*: number, NEFA: non-esterified fatty acids, IQR: interquartile range, SD: standard deviation,

1 comparison between patients and controls-interaction, Significant at the 5% level.

## Discussion

Patients with AD require lifelong glucocorticoid replacement therapy, which is most commonly provided as hydrocortisone. Endogenous cortisol production, determined by stable isotope dilution mass spectrometry in healthy individuals, is 6–11 mg/m^2^ per day [20], but the ideal hydrocortisone replacement dose remains unclear. Recommended doses vary from 30 mg of hydrocortisone to as little as 12.5 mg per day, divided in two or three daily doses [21], [22]. In general, clinicians dose hydrocortisone empirically and average doses may vary substantially between different centres or physicians. Over-replacement with hydrocortisone may result in accelerated bone loss, premature atherosclerosis and the metabolic syndrome [1], [23]–[25]. On the other hand, insufficient hydrocortisone supplementation may result in chronic fatigue and poor quality of life. Irrespective of the modality of glucocorticoid replacement patients with AD often have subjective impaired health quality [26]. A number of biochemical measures have been used to examine the adequacy or otherwise of replacement therapy in AD, but none is ideal [27]–[29]. We investigated whether determination of GCR polymorphisms could assist in individualising therapy. Patients harbouring polymorphisms that confer relative glucocorticoid resistance may, for instance, require higher doses of hydrocortisone to alleviate the symptoms of glucocorticoid deficiency completely.

We did not find any significant metabolic associations with the 9β polymorphism in AD patients in this study. Carriers of the 9β polymorphism also did not receive higher doses of hydrocortisone. Although LDLC was significantly associated with 9β status at a per-comparison level, this result did not withstand an adjustment for multiple testing. On a clinical level, determination of 9β polymorphism status is therefore of no utility in determining hydrocortisone replacement doses, however this finding needs to be confirmed in other studies. GCR variation is not the only factor that affects metabolic alterations, accounting for the multiple inconsistencies seen in various clinical settings. Earlier studies found an increased risk of cardiovascular disease, especially in the elderly in association with the 9β polymorphism and it was postulated that glucocorticoid resistance may have promoted atherosclerosis through failure to inhibit the pro-inflammatory system [11]. We were unable to replicate these findings in our study although we did not test as wide a range of variables as previous studies. We were, however, unable to demonstrate lower HDLC [30] and elevated hs-CRP [31] in relation to the 9β polymorphism to support the notion of a pro-inflammatory state.

Our study has important limitations. The 9β polymorphism was found in only 28% of the controls (41/146) and patients (39/139) respectively limiting our ability to detect genotype-phenotype associations in a study that was already limited in size by the relative rarity of AD. After correcting for multiple testing using the Holm procedure, none of our tests of association with the 9β genotype were statistically significant at the 5% level. South Africa comprises an ethnically and genetically diverse society and the heterogeneity of the study population could have obscured a true effect. Moreover, hydrocortisone doses were not standardized although the majority of patients were taking doses between 0.2 and 0.4 mg/kg. A degree of bias may have been introduced by the hydrocortisone being adjusted based on subjective and clinical parameters. Although we were not able to verify that patients took all hydrocortisone doses as instructed, these patients are educated on the importance of adherence and the risk of an Addisonian crisis. Patients with poor hydrocortisone adherence can be easily identified as they feel subjectively unwell. Lipid analyses were performed on non-fasted specimens and this may have introduced error in the calculation of LDLC according to the Friedewald equation, but TC and HDLC measurements would not have differed if samples were taken in the fasted state [32]. We also did not measure interleukin 6, which has previously been shown to be higher in those with the 9β polymorphism [11].

Ross et al have previously reported on metabolic associations with other polymorphisms in the GCR in patients with AD. The ER22/23EK polymorphism was somewhat unexpectedly associated with higher BMI, lower LDLC and higher doses of hydrocortisone. Similarly AD patients with the N363S polymorphism also received higher doses of hydrocortisone [4]. Several other studies of GCR polymorphisms have also either failed to show significant metabolic associations or have shown metabolic alterations that are difficult to reconcile with the reported changes in glucocorticoid sensitivity. For example, elderly Dutch individuals with polymorphisms that increased sensitivity to dexamethasone counter-intuitively had lower BMI and a tendency towards lower lean mass with no difference in fat mass compared to the wild type genotype [33]. The incongruity of these studies and the generally small effect sizes reported supports the notion that GCR polymorphisms may only exert minor influences on the clinical phenotype. Determining GCR polymorphisms in patients with AD is therefore not likely to be of significant clinical utility in managing patients with AD.
